# Insight into response to mTOR inhibition when *PKD1* and *TSC2* are mutated

**DOI:** 10.1186/s12881-015-0185-y

**Published:** 2015-06-17

**Authors:** Cristina Cabrera-López, Gemma Bullich, Teresa Martí, Violeta Català, Jose Ballarín, John J. Bissler, Peter C. Harris, Elisabet Ars, Roser Torra

**Affiliations:** Inherited Kidney Diseases, Nephrology Department, Fundació Puigvert, Instituto de Investigaciones Biomédicas Sant Pau (IIB-Sant Pau), Universitat Autònoma de Barcelona, REDinREN, Instituto de Investigación Carlos III, Cartagena 340-350, 08025 Barcelona, Spain; Molecular Biology Laboratory, Fundació Puigvert, Instituto de Investigaciones Biomédicas Sant Pau (IIB-Sant Pau), Universitat Autònoma de Barcelona, REDinREN, Instituto de Investigación Carlos III, Barcelona, Spain; Radiology Department, Fundació Puigvert, Barcelona, Spain; Nephrology Department, Fundació Puigvert, Instituto de Investigaciones Biomédicas Sant Pau (IIB-Sant Pau), Universitat Autònoma de Barcelona, REDinREN, Instituto de Investigación Carlos III, Barcelona, Spain; Pediatric Nephrology Department, Le Bonheur Children’s Hospital, Memphis, TN USA; Department of Biochemistry and Molecular Biology, Division of Nephrology and Hypertension, Mayo Clinic, Rochester, MN USA

**Keywords:** Tuberous sclerosis complex, ADPKD, Polycystic, mTOR inhibitors

## Abstract

**Background:**

Mutations in *TSC1* or *TSC2* cause the tuberous sclerosis complex (TSC), while mutations in *PKD1* or *PKD2* cause autosomal dominant polycystic kidney disease (ADPKD). *PKD1* lays immediately adjacent to *TSC2* and deletions involving both genes, the *PKD1/TSC2* contiguous gene syndrome (CGS), are characterized by severe ADPKD, plus TSC. mTOR inhibitors have proven effective in reducing angiomyolipoma (AML) in TSC and total kidney volume in ADPKD but without a positive effect on renal function.

**Methods and results:**

We describe a patient with independent truncating *PKD1* and *TSC2* mutations who has the expected phenotype for both diseases independently instead of the severe one described in *PKD1/TSC2*-CGS. Treatment with mTOR inhibitors reduced the AML and kidney volume for 2 years but thereafter they resumed growth; no positive effect on renal function was seen throughout. This is the first case addressing the response to mTOR treatment when independent truncating mutations in *PKD1* and *TSC2* are present*.*

**Conclusions:**

This case reveals that although *PKD1* and *TSC2* are adjacent genes and there is likely cross-talk between the PKD1 and TSC2 signalling pathways regulating mTOR, having independent *TSC2* and *PKD1* mutations can give rise to a milder kidney phenotype than is typical in *PKD1/TSC2*-CGS cases. A short-term beneficial effect of mTOR inhibition on AML and total kidney volume was not reflected in improved renal function.

## Background

Autosomal dominant polycystic kidney disease (ADPKD) is the most common kidney disorder with a Mendelian inheritance pattern, and a prevalence ranging from 1/400 to 1/1000 worldwide [1, 2]. It is responsible for 4–10 % of end-stage renal disease (ESRD) in Western countries [3, 4]. ADPKD shows both locus and allelic heterogeneity. Two causative genes—*PKD1*, and *PKD2* have been identified [5, 6]. The *TSC2* and *PKD1* genes are overlapped at their 3′ UTR ends by 3 bp. The *TSC2* gene encodes tuberin and together with *TSC1*, encoding for hamartin, causes the tuberous sclerosis complex (TSC). TSC is an autosomal dominant disorder, with high penetrance and a birth incidence of 1 in 6000–11,000 [7]. Clinically, it manifests with skin lesions, renal, neurological, pulmonary and cardiac symptoms. For the adequate diagnosis the established diagnostic criteria by Northrup et al. should be followed [8]. The clinical presentation of TSC ranges from a few features of the disease to severe neurological involvement [9].

Analysis of *TSC2* patients with severe renal cystic disease showed they can have deletions also disrupting *PKD1*; a contiguous gene syndrome (CGS). Brook-Carter et al. identified 6 *TSC2* children with very severe polycystic disease showing deletions that involved both genes [10].

These children, as well as others reported in the literature, present with enlarged polycystic kidneys recognizable *in utero*, at birth or shortly thereafter [10–12]. Their kidneys are filled by a multitude of variably sized cysts, closely resembling those seen in advanced stages of ADPKD and they usually enter ESRD in the second or third decade of life. Other typical lesions of TSC, such as angiomyolipomas (AMLs), ungual fibromas, and well-established facial angiofibromas, only appear later in life [11–17]. No description of patients having both diseases caused by two independent mutations in the *PKD1* and the *TSC2* genes have been reported to date.

Tuberin and hamartin form a complex that regulates signaling through the mammalian target of rapamycin (Rheb/mTOR/p70S6K) pathway, which controls processes such as cell growth, cell cycle progression and apoptosis. Mutations to *TSC1* or *TSC2* permit aberrant upregulation of mTOR signaling causing increased protein synthesis and cell growth [18]. Also, polycystin 1 (PC1), the *PKD1* protein product, interacts and protects tuberin S939 from AKT phosphorylation and helps to retain tuberin in the membrane to suppress mTOR activity [19–21].

Inhibition of mTOR has been proposed as therapeutic approach for both TSC and ADPKD. To date, the results are promising for TSC but are not encouraging for ADPKD [22–28].

We report here a patient with TSC and ADPKD due to independent mutations in both *TSC2* and *PKD1* who was treated with mTOR inhibitors showing a good response based on AML and cystic burden decrease but without preservation of renal function. The cross talk between tuberin, hamartin, the polycystins and mTOR are discussed to explain the phenotype of the patient and his response to mTOR inhibition.

## Methods

A 26-year-old man first presented to our renal unit at 11 years of age following detection of cystic kidneys.

**Fig. 1:**
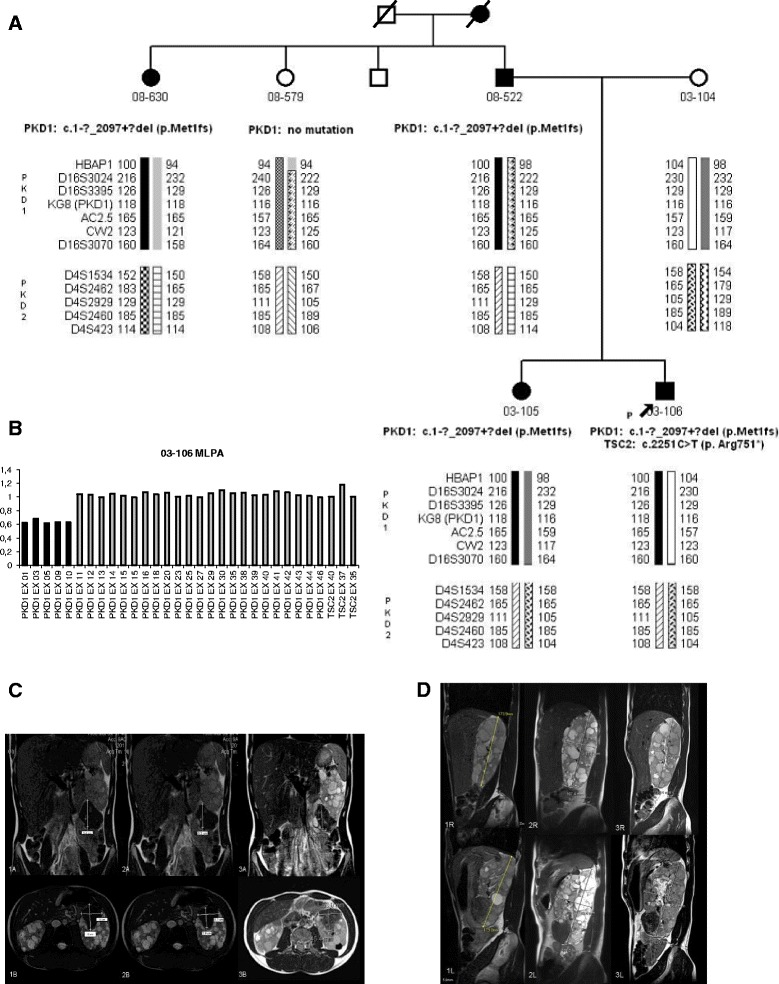
Panel **a** Pedigree of the family showing the segregation analysis of haplotypes as well as *PKD1* and *TSC2* mutations. The arrow points the proband reported in this case Panel **b** MLPA for *PKD1* gene and the 3′ end of *TSC2* gene, each bar represents the normalized peak height for the probe indicated on the x axis. The heavy black lines represent the deletion of the *PKD1* exons 1–10 in heterozygosis. Panel **c** AML volume evolution: 1A and 1B baseline; 2A and 2B at the end of 3 years treatment with mTOR inhibitors; 3A and 3B one year later (without treatment). The AML decreased in size after 3 years on treatment and slightly increased in size one year after treatment withdrawal. Panel **d** Right (top row) and left kidney (bottom row): initial MR (1R and 1L), after 3 years on treatment with mTOR inhibitors (2R and 2L) and 1 year later (without treatment) (3R and 3L)

His father, paternal aunt, paternal grandmother and sister have ADPKD (Fig. 1). The age at onset of ESRD was 68 for the grandmother, 44 for the father and 48 for the aunt. The patient’s sister has normal renal function, hypertension and enlarged kidneys (kidney length 17.5 cm) at the age of 30. There is no family history of TSC.

The patient was diagnosed with TSC at 3 months due to hypomelanic macules and a seizure. An echocardiogram revealed a cardiac rhabdomyoma, which was removed at 6 months. A brain MRI showed numerous subependymal nodules and periventricular calcifications. A retinal astrocytoma was also detected in the left eye and abnormal retinal vessels in the right one. Facial angiofibroma developed in early childhood. Development progressed normally with no further seizures or mental retardation. A kidneys ultrasound scan performed at 3 years demonstrated multiple small cysts throughout the renal parenchyma. Serial yearly ultrasound scans showed an AML of 3 cm of diameter in the left kidney at the age of 14. Cyst size and number increased along with the AML, which was 6 cm with a kidney length of 17 cm at 22 years. Because of concerns about the increasing size of the AML, local ethical approval was obtained and sirolimus started at 22 years of age (mean dose: 3 mg/day, trough levels 6.9 ± 3.8 ng/ml).

The patient and his family signed informed consents allowing researchers to publish their data and imaging. They also signed informed consent for the genetic study. The study was approved by the IRB of Fundació Puigvert.

### Imaging

Abdominal imaging evaluations were performed by 1.5 Tesla magnetic resonance (Vantage Atlas, Toshiba Medical Systems Corporation, Otawara-shi, Tochigi-ken, JAPAN) with a body phased-array coil. All studies were performed with the patient in supine position. Coronal, sagittal and axial scans were acquired with T1-weighted fast spoiled gradient echo and T2-weighted fast spin echo protocols with and without fat suppression.

Abdominal studies were analyzed by two independent radiologists with more than 10 years of experience interpreting abdominal imaging studies. Before the start of the evaluation, the radiologists showed an intra and inter-observer variability of less than 5 % in focal renal mass measurement.

The volumes of individual kidneys were measured in T1-weighted images with use of the stereologic method [25, 29, 30]. Tumour volume was estimated using a standardised validated software program (Vitrea, Vital Imaging version 4.1.14.0).

### Genetic studies

Haplotype analysis was performed using microsatellite markers within and closely flanking *PKD1/TSC2* and PKD2. The markers used were: *HBAP1, D16S3024, D16S3395, KG8, AC2.5, CW2, D16S3070* for *PKD1/TSC2* and *D4S1534, D4S2462, D4S2929, D4S2460, D4S423* for *PKD2*. The analyses of these markers were performed by PCR amplification using fluorescent primers and resolved on the ABI 3130-Avant Genetic Analyzer.

Mutation screening of *PKD1* and *TSC2* genes were performed by direct Sanger sequencing. The duplicated region of *PKD1* was amplified as five *PKD1*-specific fragments by long-range polymerase chain reaction (LR-PCR) followed by nested PCRs [31] combined with Sanger sequencing of all 46 *PKD1* exons. For *TSC2*, the 42 exons were amplified and sequenced with primers designed using genomic sequence information (GenBank accession number: NG_005895.1) and the Primer 3 (v. 0.4.0) program [32]. To screen for *PKD1/PKD2* deletion/duplication Salsa MLPA kit P351-B1/P352-B1 (MRC-Holland, Amsterdam, Netherlands) was used, which also includes probes for exons 35, 37 and 41 of the *TSC2* gene.

## Results

Six months after commencing treatment, there was a reduction in the volume of the AMLs and renal volume on MRI scan, which continued decreasing for 18 more months, although increased during the third year (Table 1) (Fig. 1c). The number of facial angiofibromas remained unchanged, but the lesions were smaller, paler and less rough. At that time, the negative results of two large trials using mTOR inhibitors in ADPKD were released, which together with the evidence of regrowth under treatment, prompted us to discontinue the mTOR inhibition.

**Table 1:** Progression of kidney and angiomyolipoma volume along time. Laboratory test while on and off mTOR inhibitor treatment

	ᅟ

Shaded columns represent the period in which the patient received mTOR inhibitors

One year after discontinuation, the AML had further increased along with the renal volume, and the GFR decreased (Table 1). The only side effect of the drug was an increase of the protein to creatinine ratio during treatment which decreased after cessation (Table 1). Although normal blood pressure was recorded while on treatment, when it stopped a slight but abnormal increase in diastolic BP was detected and treatment with ACEI was started achieving a good control of BP.

Initially linkage analysis showed that the ADPKD family was linked to the *PKD1* gene (Fig. 1). Also, heterozygosity of the KG8 microsatellite marker (located in the 3′ region of *PKD1*) ruled out a contiguous gene syndrome. Subsequently, sequencing and MLPA analysis of the *PKD1* and *TSC2* genes, disclosed a deletion of *PKD1* (exons 1–10, Fig. 1b): c.1-?_2097 + ?del, (p.Met1fs) (definitely pathogenic mutation), and a *TSC2* nonsense mutation: *TSC2* c.2251C > T (p.Arg751*) (definitely pathogenic mutation). The *PKD1* mutation was present in all affected members of the family while the *TSC2* mutation was absent in all but the proband.

## Discussion

A single individual carrying mutations in two different genes causing two different diseases, and therefore suffering both entities, is not frequent but depends on the prevalence of each disease. Taking into account that the prevalences of ADPKD and TSC are around one in 800 and one in 8.000, respectively, the probability for the simultaneous occurrence of these two diseases is approximately one in 6.400.000 births. Due to difficulty of genetic testing to demonstrate the coexistence of mutations in the two genes, probably this unusual population has been under represented in the literature.

Most patients with TSC and severe cystic disease have a CGS with a deletion involving the coding regions of both the *TSC2* and the *PKD1* genes (*PKD1/TSC2*-CGS) [10, 14, 33, 34]. They consist of ~3 % of TSC patients overall [9]. The prevalence of kidney cysts in TSC alone is between 30 and 50 %, more frequent in *TSC2* than *TSC1* [9, 26, 35], especially when the *TSC2* gene harbours a nonsense or frameshift mutation [36]. But these non-CGS TSC patients generally have just a few small cysts of little clinical relevance [17, 37] and, do not present renal failure [37]. However, children with *PKD1/TSC2*-CGS usually enter ESRD in the second or third decade of life [10, 17]. Nevertheless, some *PKD1/TSC2*-CGS patients have milder renal disease [13, 38] with mosaicism being the main reason which is rather common in these cases but not supported by the blood derived DNA studies here [12, 17, 37].

A case with separate *TSC1* and *PKD2* mutations had a very modest cystic phenotype, probably due to the relatively mild disease in *PKD2* compared to *PKD1* and because *TSC1* mutations are rarely associated with renal cysts [39].

The case we present here has a *de novo* nonsense mutation in the *TSC2* gene and a germ line deletion of the first 10 exons of the *PKD1* gene. The *TSC2* mutation appeared spontaneously in this patient and does not show mosaicism in blood but we cannot rule out mosaicism at the organ level, particularly in the kidney. ADPKD in this family is quite severe but what is most surprising is the apparent limited impact of an additional *TSC2* mutation in the proband. He will probably enter ESRD sooner than his relatives but not by many years. In fact, his kidney size does not differ significantly from his sister. If the CGS phenotype were just due to an additive effect of disrupting both genes, one would expect the same effect of a truncating mutation in both genes than a deletion involving both of them. However, as some cases of late onset of ESRD have been described in *PKD1/TSC2*-CGS the real explanation for a milder phenotype remains elusive [5, 13, 38]. Given that the primary transcripts from these genes slightly overlap at the 3′ region, regulation due to antisense noncoding (nc)RNAs or miRNA binding could be unusually altered. One limitation of the present case is the impossibility to know whether the mutations are *in cis* or *in trans* due to the fact that the *TSC2* mutation is *de novo*. If the *TSC2* mutation is in cis position with the *PKD1* deletion, the proband is expected to have similar disease presentation as patients with *TSC2/PKD1* CGS. However, if the two mutations are *in trans*, the second hit, likely by loss of heterozygosity, can only lead to loss of both *TSC2* and *PKD1* function on one chromosome while leaving one normal copy of both. Under this scenario, the proband will maintain normal function of one of the two genes with a milder phenotype than expected for *TSC2/PKD1* CGS. However, as stated by Brook Carter et al., the phenotypes associated with independent deletion of each of the contiguous genes are different and the contiguous syndromes represent an accumulation of these phenotypes [10].

Tuberin, hamartin and PC1 are located at the basal body or primary cilia and have been proposed to form a complex which inhibits mTOR [20]. Alternatively, the CGS phenotype may be due to disruption of two independent cystogenic mechanisms, PC1 involving cilia and tuberin through proliferation. Hartman found that Rapamycin enhanced cilia formation in *TSC1* and *TSC2* null cells and concluded that the efficacy of mTOR inhibitors on renal cystic disease in patients carrying a TSC mutation or *PKD1/TSC2*-CGS may differ from its efficacy in ADPKD [40]. The fact that some cysts in ADPKD tissue and *Pkd1* mutant kidneys do not appear to upregulate mTORC1 and the small number of cysts in patients with TSC calls into question the essential role of the mTOR cascade in cyst formation [41]. Trials have demonstrated efficacy of mTOR inhibitors in TSC with clear reduction in the volume of AML [23–26]. However, mTOR inhibitor studies in ADPKD patients have not been successful [27, 28]. In AML, reduction of volume and probably vascularisation is an excellent end point, but for ADPKD a positive impact on renal function as well as a reduction in kidney volume is ideally required. This is the first described case treated with mTOR inhibitors having TSC and ADPKD and showed that mTOR inhibition reduced AML volume and cystic volume during the first 2 years, but renal function still declined. Interestingly, both AML and renal volume increased during the third year even while on the treatment with adequate Rapamycin plasma levels, which may be due to an adaptive escape mechanism from mTOR inhibition, although this is an uncommon event in non-cancer cells. This patient experienced an increase in the protein/creatinine ratio which may be explained by the effect of mTOR inhibition. The exact mechanism by which mTOR inhibitors affect glomerular permeability is not known. Many mechanisms have been propose such as, decreased VEGF synthesis and expression, dose-related alteration of podocyte slit diaphragm-associated protein structure and activation of the innate immune system, resulting in an increased number of glomerular macrophages [42–47].

Longer term trials in *PKD1/TSC2*-CGS patients would be interesting in light of our results and also it would be interesting to test the levels of mTOR activation in the peripheral blood of classical CGS patients and of this specific case.

## Conclusions

Although *PKD1* and *TSC2* are adjacent genes and tuberin-hamartin and PC1 may cross-talk and regulate mTOR inhibition, having independent mutations in *TSC2* and *PKD1* does not necessarily give rise to the typically severe *PKD1/TSC2*-CGS phenotype. However this is a unique case and the mild disease presentation needs further verification when additional cases become available.

mTOR inhibitors are efficient in reducing AML and ADPKD kidney volume, but do not have a positive impact on renal function.
